# Supplementary material for: The interactome of intact mitochondria by cross-linking mass spectrometry provides evidence for coexisting respiratory supercomplexes
Source: Mol Cell Proteomics. 2017 Dec 8;17(2):216–32. doi: 10.1074/mcp.RA117.000470 (PMC5795388; doi:10.1074/mcp.RA117.000470)
Supplement: Supplemental Data [file supp_RA117.000470_133922_0_supp_23978_fzffwf.zip › spectra_annotation/mito_spectra_annotation/443-1-2-1-1-1.pdf]

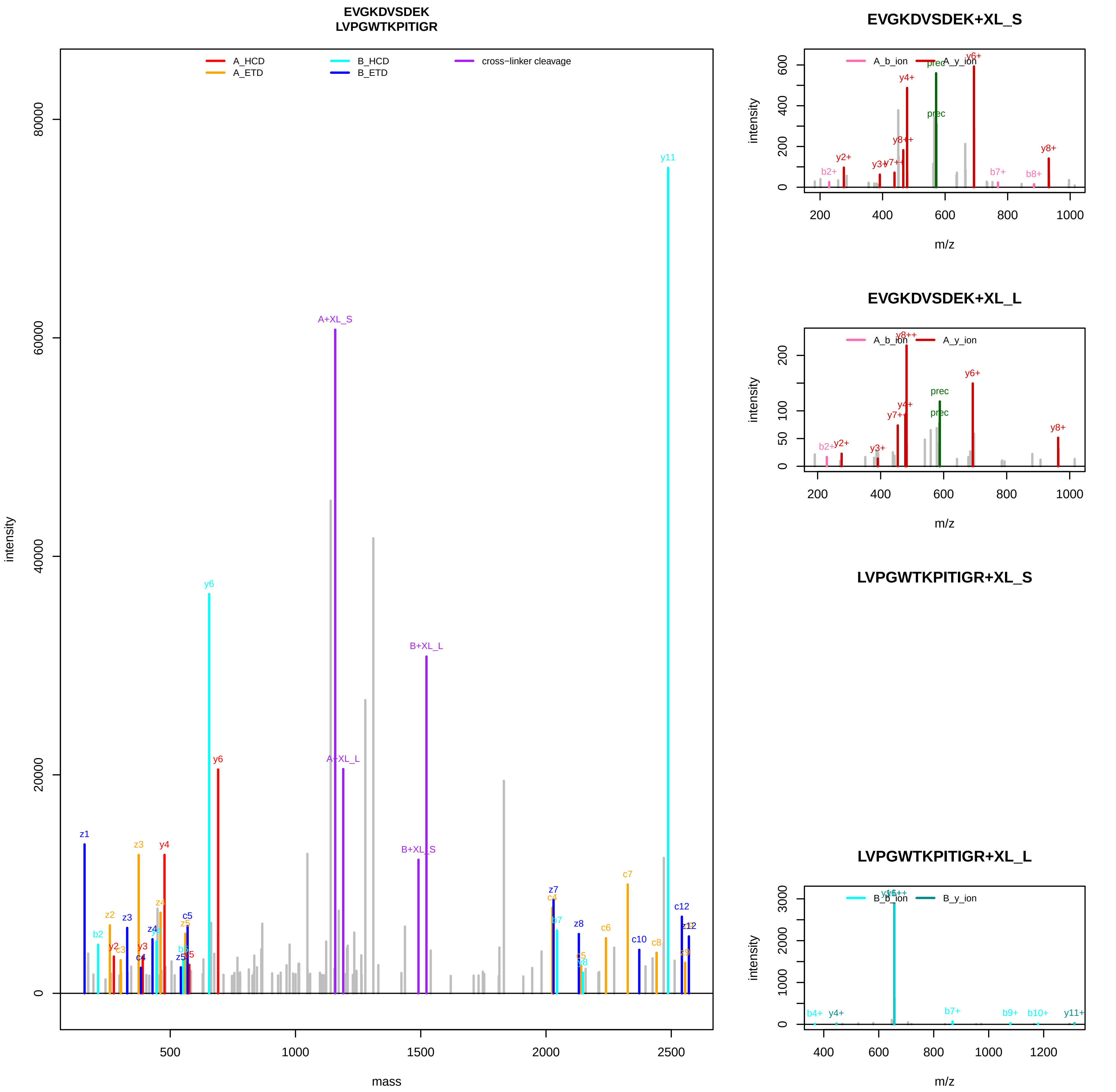

EVGKDVSDEK
LVPGWTKPITIGR
A_HCD
A_ETD
B_HCD
B_ETD
cross−linker cleavage
intensity
mass
0
20000
40000
60000
80000
500
1000
1500
2000
2500
y11
A+XL_S
y6
B+XL_L
y6
A+XL_L
B+XL_S
z1
z3
y4
z2
b2
z3
y2
c3
y3
c4
z4
z4
z5
c5
z5
b5
y5
z7
c4
b7
z8
c5
y8
c6
c7
c10
c8
c12
z12
z9
EVGKDVSDEK+XL_S
EVGKDVSDEK+XL_L
LVPGWTKPITIGR+XL_S
LVPGWTKPITIGR+XL_L
A_b_ion
A_y_ion
B_b_ion
B_y_ion
prec
y6+
y4+
y8++
y2+
y3+
y7++
b2+
b7+
b8+
y8+
y4+
y16++
b4+
b7+
b9+
b10+
y11+
m/z
